# A Hybrid PSO–SVM Model Based on Safety Risk Prediction for the Design Process in Metro Station Construction

**DOI:** 10.3390/ijerph17051714

**Published:** 2020-03-05

**Authors:** Ping Liu, Mengchu Xie, Jing Bian, Huishan Li, Liangliang Song

**Affiliations:** 1 School of Civil Engineering, Lanzhou University of Technology, Lanzhou 730050, China; liupvip@foxmail.com (P.L.); xmc1104@126.com (M.X.); lhs1968lut@163.com (H.L.); 2 School of Management Science and Real Estate, Chongqing University, Chongqing 400045, China; 3 Institute of Engineering Management, Hohai University, Nanjing 211100, China; liang.liang.song@hotmail.com

**Keywords:** safety risk prediction, metro station construction, design for safety, support vector machine, particle swarm optimization

## Abstract

Incorporating safety risk into the design process is one of the most effective design sciences to enhance the safety of metro station construction. In such a case, the concept of Design for Safety (DFS) has attracted much attention. However, most of the current research overlooks the risk-prediction process in the application of DFS. Therefore, this paper proposes a hybrid risk-prediction framework to enhance the effectiveness of DFS in practice. Firstly, 12 influencing factors related to the safety risk of metro construction are identified by adopting the literature review method and code of construction safety management analysis. Then, a structured interview is used to collect safety risk cases of metro construction projects. Next, a developed support vector machine (SVM) model based on particle swarm optimization (PSO) is presented to predict the safety risk in metro construction, in which the multi-class SVM prediction model with an improved binary tree is designed. The results show that the average accuracy of the test sets is 85.26%, and the PSO–SVM model has a high predictive accuracy for non-linear relationship and small samples. The results show that the average accuracy of the test sets is 85.26%, and the PSO–SVM model has a high predictive accuracy for non-linear relationship and small samples. Finally, the proposed framework is applied to a case study of metro station construction. The prediction results show the PSO–SVM model is applicable and reasonable for safety risk prediction. This research also identifies the most important influencing factors to reduce the safety risk of metro station construction, which provides a guideline for the safety risk prediction of metro construction for design process.

## 1. Introduction

Urban rail transit construction is very significant in promoting urban economic development. Urban metros are developing rapidly around the world since they are a fast, efficient, safe and comfortable transportation mode [1]. At the end of 2018, 35 cities in mainland China had constructed 185 urban rail operation lines with a total length of 5761.4 km according to the Annual Urban Rail Transit Statistical and Analysis Report [2]. The scale of lines planned and under construction has been growing steadily. In addition, the annual completed construction investment has reached a new record. However, with the rapid development of the metro, construction safety accidents occur frequently, which cause a large number of casualties and economic losses [3]. According to statistics, during the period from 2002 to 2016 (statistics to March 2016), 246 accidents occurred in metro station construction in China, of which metro station projects accounted for 57% [4].

Natural science is concerned with explicating and understanding natural phenomena, while design science attempts to create things that serve human purposes [5]. Design science is accomplished through knowledge-based pattern recognition and problem-solving search processes [6]. Meanwhile, building and evaluating are design science research activities aimed at improving performance [7]. Such pattern recognition activities are an important part of human cognition and are fairly central to scientific reasoning as well [8]. The roots of the design science paradigm are in engineering and the sciences of the artificial [7]. As mentioned above, design science is essentially a problem-solving process and a key activity in fields of architecture, engineering and urban planning [9]. In the field of engineering, the International Labor Organization pointed out that about 60% of engineering accidents were related to design [10]. Gambatese [10] analyzed the causes of 100 construction accidents and found that in 47% of cases, the probability of accidents can be reduced by improving the design scheme. In line with this view, the National Institute for Occupational Safety and Health put forward Prevention through Design [11], which considered the needs of occupational health and safety in the design process. Moreover, Design for Safety (DFS) is one of the most effective ways to consider safety risks in the design process [12], which is also considered as an important way to achieve social sustainable development. This is because it can not only reduce the safety risk and improve the level of safety management, but also can reduce the risk of construction-period extension and costs caused by safety accidents [12]. Thus, it is beneficial to utilize the DFS to enhance the safety of metro station construction.

DFS has different definitions and expressions in the existing literatures, such as Design for Safety [13], Safety through Design [14], Prevention through Design [11], Design for Construction Safety [15] and Construction Hazard Prevention through Design [16]. In summary, DFS is defined as eliminating or avoiding dangerous sources through standardized design and improved design to achieve the purpose of reducing safety accidents, which considers the safety and health of construction, operation and maintenance personnel, and project end-users in the early design and planning process of a project. Many researchers have found that design work has contributed to safety construction. By a comparison of the causes of construction accidents with other factors, Suraji et al. [17] found that planning and design work are the proximal factor resulting in improper site conditions and construction operations. Haslam et al. [18] put forward the hierarchy of causal effects of construction accidents, and found that design work is the critical factor causing accidents. Furthermore, designers can alleviate safety accidents by choosing alternative technologies and improving project resilience [19]. Yuan et al. realized that integrating the DFS knowledge base and Building Information Modelling (BIM) can evaluate safety risks during the design phase [20]. In addition, Szymberski [21] proposed the time-safety impact curve, and found that the ability to affect life cycle safety will reduce as the project proceeds. Meanwhile, improving designers’ hazard identification skills were thought to be an urgent need [22].

The existing research into DFS mainly focuses on three aspects: (1) research on the basic theory of DFS, which includes the analysis of safety risk sources caused by design [23], the analysis of the correlation between design and safety accidents [24] and the promotion of sustainable improvement by DFS [25]; (2) the analysis of the obstacles and driving factors of the development of DFS, for which the main obstacles include the lack of safety design-related tools and standards [26,27], insufficient knowledge reserve of designers [28], increased design costs caused by DFS [27,29]; and (3) research on the practical application of DFS; for instance, Gambatese et al. [30] built the first “design for construction safety toolbox” based on the safety recommendation database in 1997. The toolbox links the design and construction process to help designers identify the safety risks of specific project construction. Subsequently, the safety design tool based on the matching relationship between safety risk and design scheme was developed. Gambatese and Hinze [26] added safety manuals and safety guides to the toolbox. Hadikusumo and Rowlinson [31] built similar safety design process tools. Seo and Choi [32,33,34,35,36,37,38,39,40,41,42,43] evaluated and selected the design schemes of specific metro engineering projects by establishing the matching relationship between design suggestions and safety risks.

The risk prediction of metro station construction can provide a guideline for the implementation of DFS. On one hand, the identification of safety risk influencing factors is one of the most significant procedures in the risk-prediction process [44]. In such a case, the identification of safety risk influencing factors for the metro construction process has attracted much attention. For example, Yu et al. [45] analyzed the influencing factors of safety management in metro construction, and pointed out that safety attitude, construction site safety, government supervision, market restrictions and the unpredictability of tasks were the most important factors. Zhang et al. [46] classified the causes of construction accidents in the Beijing metro and found that inadequate management was the biggest cause of accidents, but the most serious accidents were caused by leaks or fractures in pipes and poor geological conditions. Ghosh and Jintanapakanont [47] separated and evaluated the key risk factors in the Thailand metro project, obtaining nine key factors and 35 sub-key factors by using the key factor analysis method. On the other hand, the construction of a prediction model is another key procedure in the risk-prediction process. Thus, many scholars have focused on the construction of prediction model. For instance, Wu et al. [48] presented a systemic Bayesian network method for the dynamic risk analysis of adjacent buildings in tunneling environments. Li et al. [49] proposed the safety risk identification system and early warning system for China’s metro construction based on BIM. Zheng et al. [50] used the fuzzy analytic hierarchy process (AHP) and the comprehensive evaluation method to assess the metro construction risk of Changchun No.1 in China.

However, as mentioned above, there are some limitations in the existing literature: (1) few studies have focused on the leading role of design in metro construction, and few researchers have incorporated risk prediction into the DFS to enhance the safety of metro station construction. The design result is the most important work basis for the field operator. If there are construction safety risks in the preliminary design documents and these are not effectively dealt with, these defects will lead to an unsafe state of objects (including the environment) and the unsafe behavior of field operators in the construction process [51]; (2) some traditional risk-prediction methods, such as the neural network method, fuzzy comprehensive evaluation method, Bayesian network, and so on, have some shortcomings, such as low accuracy and low prediction efficiency [52]. In recent years, the support vector machine (SVM) model based on particle swarm optimization (PSO–SVM) has been widely used in many fields, which can overcome these problems [53,54]. For example, Zhou et al. [55] built the prediction model of PSO–SVM to predict the landslide displacement, and demonstrated that the proposed PSO–SVM model can better represent the response relationship between the factors and the periodic displacement. Chen et al. [56] used the evaluation model of short-term atmospheric pollutant concentration forecasting based on PSO-SVM, which demonstrated the superior performance of the proposed hybrid model. However, little attention has been paid to developing a hybrid risk-prediction framework for metro station construction by using the PSO–SVM model.

Design science is considered as practical knowledge used to support design activities, which seeks various approaches to a real-world problem of interest to practice [57,58]. Improving the ability of identifying safety risks is an available method to promote the implementation of DFS at the design phase of engineering project [59]. Therefore, this paper aims to construct a safety risk-prediction model to improve the performance of DFS in practice based on PSO–SVM. The PSO–SVM intelligent prediction model is used to predict the safety risks of a specific metro station construction project. The remainder of this paper is presented as follows. Section 2 introduces the basic principle and analysis method of PSO–SVM. Section 3 constructs the framework for the safety risk prediction of metro station construction. Section 4 applies the hybrid model to predict the safety risk of a case study of metro station construction. Finally, conclusions are presented in Section 5.

## 2. Methodology

### 2.1. Support Vector Machine

The SVM [60] is a machine learning method based on the extended development of statistical learning theory [61,62]. The basic idea of the theory is to use non-linear mapping to project data points from a low-dimensional space into a high-dimensional space and linearly regress them in the high-dimensional feature space [53].

Machine learning is mainly divided into supervised learning and unsupervised learning [63]. Supervised learning usually requires a fully annotated training set to train a model which can be generalized to other unseen data. Unsupervised learning applies to datasets which only have input features but are missing annotations. In this research, feature attributes and annotations are both provided, and a machine learning model of metro construction safety risk is trained by analyzing the relationship between influencing factors and safety risks. Therefore, this paper addresses the problem of prediction of safety risk of metro construction engineering from a novel machine-learning perspective. Scholars have proposed some machine-learning methods, including neural network, artificial neural network (ANN), back propagation (BP), decision tree and SVM, etc. Traditional neural networks (NN) face some problems related to convergence and local optimization [56]. Moreover, the defects of faulty theory foundation, local minimum and over fitting weakened the ability of prediction [64]. Meanwhile, ANN shows a promising performance in fitting non-linear variables, but the complex relationships amongst some variables can affect its performance [65]. Back propagation (BP) is widely used in neural network models, which are trained by the error back propagation algorithm. The convergence speed of the back propagation neural network is slow, and it cannot guarantee the convergence to the global optimum. To address these problems, SVM is used for machine learning given its excellent performance in dealing with the statistical learning theory for small sample and in addressing global optimization and the principle of structural risk minimization [56].

**Figure 1:**
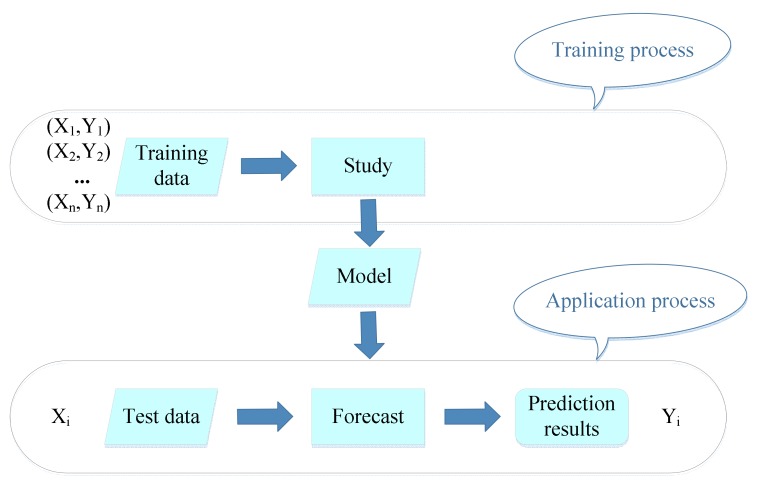
The process of supervised learning algorithms.

A successful supervising learning algorithm usually contains two stages: one is the training stage, and the other is the practical application stage. The purpose of supervised learning training is to analyze the dependency $x_{i}\to y_{i}$ between the input and target according to the given training sample $\left(x_{1},y_{1}\right),\left(x_{2},y_{2}\right),\ldots ,\left(x_{n},y_{n}\right)$. Assuming the evaluation function is $f:x\to f\left(x\right),y^{\prime }=f\left(x\right)$, the output $y^{\prime }$ is the target classification based on the evaluation function, as shown in Figure 1.

Supposing there are N samples in the dataset space, ${\left(x_{{}_{i}},y_{i}\right)}_{1\leq i\leq N}$ as training samples. The input variables are mapped into a high-dimensional linear feature space through a non-linear transformation. Then the optimal decision function is constructed. The dot product operation in the higher dimensional feature space is replaced by the kernel function in original space, and the global optimal solution is obtained by the training of the finite sample. Equation (1) represents the classification hyperplane.

(1) $$y_{i}(\omega \cdot x_{i}+b)\geq 1$$

where, ω is the weight vector; *b* is bias; “·” is the inner product; $x_{i}$ is a D-dimensional real input vector; $y_{i}$ represents the corresponding annotation of $x_{i}$, $y_{i}=\pm 1$ which is represented as precipitation occurrence or not here [66].

In order to maximize the interval, one only needs to calculate $\frac{1}{2}\omega^{T}\omega$. The basic type of SVM is as shown in Equation (2):

(2) $$\left\{\begin{array}{l} \underset{\omega ,b}{\min}\frac{1}{2}\omega^{T}\omega \\ subject\;to\;y_{i}\left(\omega^{T}\cdot x_{i}+b\right)-1\geq 0 \end{array}\right.$$

where i=1,2,⋯,N. The basic type of the above problem is a constrained convex quadratic programming problem. In order to solve dual problems, the Lagrange function is used to fuse the constraint into the objective function. It can be defined as Equation (4):

(3) $$\underset{a}{\max}\sum_{i=1}^{N}\alpha_{i}-\frac{1}{2}\sum_{i=1}^{N}\sum_{j=1}^{N}y_{i}y_{j}\alpha_{i}\alpha_{j}\left(x_{i}\cdot x_{j}\right)$$

subject to:

(4) $$\left\{\begin{array}{l} \sum_{i=1}^{N}\alpha_{i}y_{i}=0\\ 0\leq \alpha_{i}\leq C,\;i=1,2,\cdots ,N \end{array}\right.$$

where $\alpha_{i}$ is a Lagrange multiplier for each training sample, the sample is the support vector for which $\alpha_{i}=0$, lying on one of the two hyper-planes: $\left(\omega \cdot x^{+}\right)+b=+1$; $\left(\omega \cdot x^{-}\right)+b=-1$.

According to the Karush–Kuhn–Tucker condition, the optimization problem must satisfy the last one in Equation (4) [67]. When dealing with the non-linear SVM problem, SVM introduces a kernel function to map relationship of the training samples from the original space to the high-dimensional space:

(5) $$\underset{\alpha }{\max}\sum_{i=1}^{N}\alpha_{i}-\frac{1}{2}\sum_{i=1}^{N}\sum_{j=1}^{N}y_{i}y_{j}\alpha_{i}\alpha_{j}K\left(x_{i},x_{j}\right)$$

The $K\left(x_{i},x_{j}\right)$ is the kernel function of SVM that represents the inner product of two vectors [68]. It is defined as $K\left(x_{i},x_{j}\right)=\varphi \left(x_{i}\right)\cdot \varphi \left(x_{j}\right)$. The radial basis function (RBF) is one of the most popular kernel functions, and the RBF kernel function is accounted for in the non-linear problems in this paper:

(6) $$K\left(x_{i},x_{j}\right)=\exp\left\{-g∥x_{j}-x_{i}{∥}^{2}\right\}$$

where g is the kernel parameter to measure the width of kernel function in RBF.

Therefore, there are two important parameters affecting the learning performance of SVM that are the penalty parameter c and the kernel function parameter g, which can affect the classification accuracy of the SVM. It is necessary to utilize optimization algorithm to optimize the parameters of SVM.

### 2.2. The Multi-Classification Support Vector Machine (SVM) Prediction Model Based on Binary Tree

**Figure 2:**
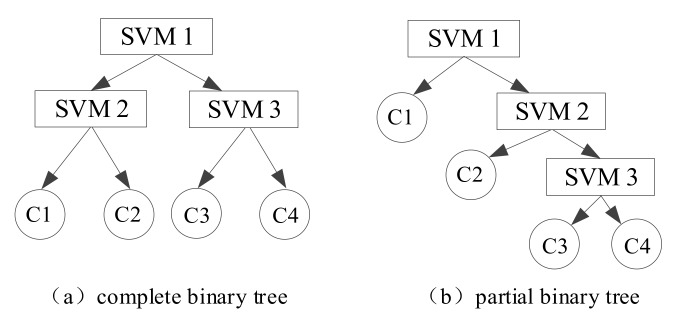
Complete (**a**) and partial (**b**) support vector machines (SVMs).

The classic SVM is mainly designed for binary classification problems and cannot be directly used for multiclass classification problems. However, there are several levels of safety risk prediction for metro construction, and the binary classifier cannot meet the requirements of prediction. Therefore, this study uses the binary tree to solve multiclass classification problem. The principle is to divide all categories into two sub-classes by constructing a binary tree and using a single SVM to do binary classification each time; then a sub-class is divided into two sub-sub classes, which continues until all nodes contain only a single category. For N class classification problems, the method needs N−1 binary classifiers to distinguish from the SVM of the root node in turn. According to the specific classification problem, binary tree algorithms can be divided into complete binary trees and partial binary trees [69], as shown in Figure 2. In Figure 2b, the sorting order of the classifier in the partial binary tree is 1, 2, 3, and 4; since there are 4 categories, corresponding to 4 structures respectively.

In the partial binary tree structure, we assume that the correct rate of each layer is $p_{1},p_{2},\ldots ,p_{k}$. If the correct rate of class division is 1, the classification accuracy of all categories is:

(7) $$\left\{\begin{array}{l} SVM_{1}=p_{1}\\ SVM_{2}=p_{2}\\ \;\;\;\vdots \\ SVM_{k-1}=SVM_{k}=p_{1}\times p_{2}\times \cdots \times p_{k} \end{array}\right.$$

From Equation (7):

(8) $$SVM_{1}>SVM_{2}>\cdots >SVM_{k-1}=SVM_{k}$$

**Figure 3:**
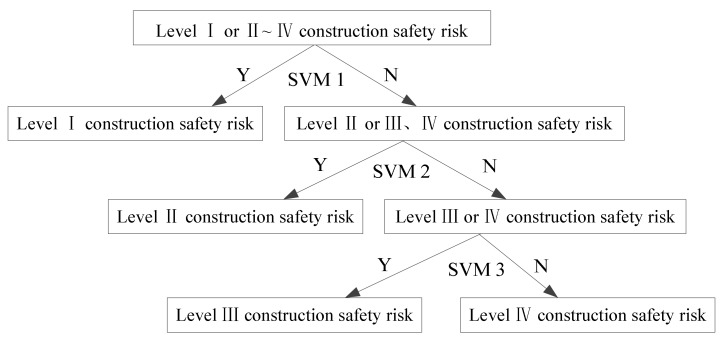
The prediction model of the SVM metro construction safety risk classification binary tree.

It can be found from Equation (8) that the deeper the binary tree classifier is, the lower the recognition accuracy. Only by making the shallow SVM recognize correctly can improve the performance of the deep SVM. Therefore, in the binary tree structure, the classification nodes usually play a more important role. According to this fact, a safety risk classification and prediction model based on a binary tree SVM is designed, as shown in Figure 3.

It can be found from Figure 3 that the safety risk level of metro construction is high and is in the upper node. The structural design of this binary tree classifier is mainly based on two aspects: (1) the higher the safety risk level of the metro construction, the greater the loss caused by an accident. However, the recognition accuracy of the binary tree classifier decreases with the increase of depth; therefore, it is a priority to identify the level I safety risk, which is the most critical and has the largest loss, to ensure the accuracy of identification.

The prediction model of SVM metro construction for the safety risk classification of the binary tree includes three binary classifiers of SVM, and each SVM classifier determines a category: (1) training the predictive model—firstly, the collected construction safety risk samples of Class I are taken as the positive class of the first sub-classifier, which are identified as 1. Then, the remaining three types of sample sets are combined as the negative class of the first sub-classifier, which are identified as –1, and the training classifier is SVM 1; (2) the second-level construction safety risk sample from the remaining three types of samples is selected as the positive class of the second sub-classifier, whose category is identified as 1, and the remaining class II and class III samples are combined as the negative class of the second sub-classifier, which are identified as –1, and the training classifier is SVM 2; (3) the third sub-classifier SVM 3 is established to complete the construction of the binary tree SVM.

### 2.3. Parameter Optimization of SVM Model Based on Particle Swarm Optimization (PSO)

The SVM model has a good ability to solve small-sample, high-dimensional and non-linear problems. However, the choice of kernel function parameter g and the penalty parameters c of the SVM model have important influences on the accuracy of the SVM model. The different types of kernel functions determine the different properties of the SVM model. In the present work, kernel functions, such as linear kernel, polynomial kernel, sigmoid function and RBF are commonly used for SVM modeling [70]. With the wide convergence domain, the RBF has the advantage of being able to approximate an arbitrary non-linear and high-dimensional computation function, so it is the most widely used kernel function. Besides, RBF is a prior selection, since it effectively reduces complexity for inputs by only adjusting c and g [71].

In the SVM–RBF model, the appropriate model parameter setting has a heavy impact on the classification accuracy of the SVM model [72]. The penalty parameter c represents a “degree of punishment” that controls the sampling error. If the value of c is large, model may suffer from overfitting problem, that is model can fit training data well but perform poorly on other unseen data. If the value of c is small, the complexity of the model is reduced, and the model’s generalization ability may be improved but may also suffer from underfitting problems. The parameter g of the kernel function represents the width of the RBF kernel function, and the larger value of g, the higher correlation between the support vectors. Therefore, c and g affect the performance of SVM together. Only by constantly adjusting the model parameters to achieve the best combination of model parameters can the SVM machine-learning ability and regression prediction effect be improved. Therefore, the penalty parameters *c* and kernel parameters *g* should be optimized.

The traditional methods of SVM parameter selection are generally the cross validation method and grid search method. These two methods have some limitations of low efficiency, low precision, and the search parameters cannot be optimized. PSO has the advantages of a simple algorithm structure, such as high precision, fast convergence speed and strong global search ability. PSO is an algorithm developed in recent years, and it was inspired from the feeding behavior characteristic of a bird flock, which is used for solving optimization problem. PSO was first proposed by Kennedy and Eberhart [73]. In PSO, each particle represents a potential solution to the problem. The common feature of the particle is represented by position, speed and fitness value. Each particle updates the position and speed in the next iteration by tracking the fitness extreme value. The fitness extreme value mainly includes the individual extreme value $P_{best}$ and the global extreme value $g_{best}$. The position of the particle’s previous best performance in a vector called $P_{best}$, and the $g_{best}$ value is tracked by the particle swarm optimizer. The fitness value can be calculated through the fitness function, which can estimate the merit of the particles. After discovering $P_{best}$ and $g_{best}$, PSO identifies the speed and distance of each particle [56].

PSO has the advantages of a simple algorithm structure, such as high precision, fast convergence speed and a strong global search ability. It is widely used in data optimization and data mining. This research adopts the PSO to search and optimize the kernel parameters and penalty factors of the SVM model instead of the traditional parameter optimization method [74]. By constructing the PSO–SVM prediction model, the learning ability and prediction effect of the safety risk-prediction model of metro construction are improved.

In the D–dimensional space, the space vector $X_{i}={\left(X_{i1},X_{i2},\cdots ,X_{iD}\right)}^{T}$ is represented as the i−th particle, where i=1,2,⋯,n, $X_{i}$ is the position of the ith particle and a possible solution.

The velocity and position of the particles are iterated to obtain the equation as follows [75]:

(9) $$\left\{\begin{array}{l} V_{id}^{k+1}=\omega V_{id}^{k}+c_{1}r_{1}\left(P_{id}^{k}-X_{id}^{k}\right)+c_{2}r_{2}\left(P_{gd}^{k}-X_{id}^{k}\right)\\ X_{id}^{k+1}=X_{id}^{k}+V_{id}^{k+1} \end{array}\right.$$

where: k is the k−th iteration; $V_{i}=\left(V_{i1},V_{i2},\cdots ,V_{iD}\right)$ is the velocity of the i−th particle, and $P_{i}=\left(P_{i1},P_{i2},\cdots ,P_{iD}\right)$ is the optimal position of this particle. The optimal swarm position is $P_{g}=\left(P_{g1},P_{g2},\cdots ,P_{gD}\right)$. Under the condition of the i−th particle at the k−th iteration, $X_{id}^{k+1}$ and $V_{id}^{k+1}$ are the d−th location and speed component. Parameters $c_{1},c_{1},r_{1}$, and $r_{2}$ are the random number, the range is 0 to 1, and ω is the inertial weight of the PSO algorithm.

The process of using PSO parameters for optimization is as follows:

**(i)** The population is initialized. The population size, the maximum number of iterations of the population, the penalty factor c and the optimization range of the kernel parameter g are set. The learning factors $c_{1}$ and $c_{2}$ are adopted by the linear learning strategy. The inertia weight ω is adopted by the linear decreasing strategy.

**(ii)** The position $x_{i}^{0}$ and velocity $v_{i}^{0}$ of the initial particles within the allowed range are generated randomly.

**(iii)** Fitness calculation: the fitness value is the mean squared error (MSE) when cross-checking the training set.

**(iv)** The fitness value $f_{i}$ of the current position of each particle in the population with the individual extreme value $P_{best}$ is compared; if $f_{i}<P_{best}$, then $P_{best}=f_{i}$, otherwise $f_{i}$ remains unchanged.

**(v)** The individual optimal value $P_{best}$ of each particle in the population with the population global extremum is compared; if $P_{best}<g_{best}$, then $g_{best}=P_{best}$, otherwise $g_{best}$ remains unchanged.

**(vi)** If the termination condition is satisfied, the iteration is stopped and the positional parameters of the optimal particle are output, that is, the optimal penalty coefficient c of the SVM and the kernel function parameter g are output, otherwise steps iii–vi are repeated.

## 3. Framework for Safety Risk Prediction of Metro Station Construction

**Figure 4:**
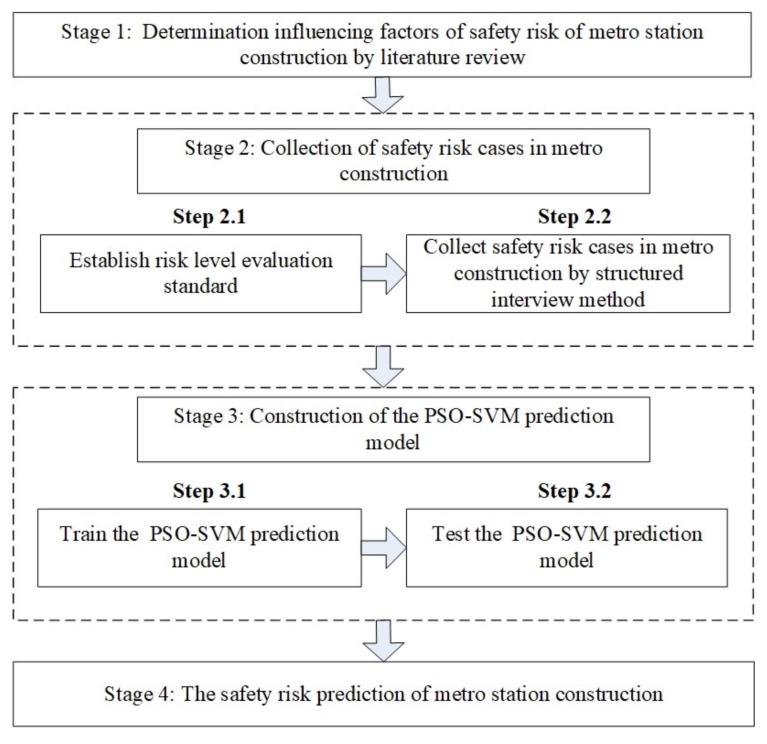
The overall framework in this study.

In this section, a hybrid risk-prediction framework for metro station construction is presented by using the PSO-SVM prediction model. The flowchart of this framework is presented in Figure 4. First, the literature review and code of construction safety management analysis methods are used to identify the influencing factors of safety risk in metro station construction. Second, a structured interview is used to collect safety risk cases of metro construction projects. Then, the PSO–SVM model is constructed to predict the safety risk. Finally, the proposed framework is applied to a case study of metro station construction.

### 3.1. Stage 1: Identify the Influencing Factors of Safety Risk in Metro Construction

In order to obtain the influencing factors of metro construction safety risk more comprehensively, the relevant core influencing factors are selected from the existing construction safety management standards, specifications and literature analysis.

### 3.2. Stage 2: Collection of Safety Risk Cases in Metro Construction

Due to the complexity of the metro construction process, the related safety risk influencing factors frequently cannot be measured directly; the research data can only be obtained indirectly. Therefore, an expert interview method is adopted to collect construction safety risk cases. The expert interview is mainly divided into structured interviews and semi-structured interviews. The difference mainly lies in the degree of the researcher’s control over the interview process. Structured interviews usually use questionnaires which are uniformly designed and structured, while semi-structured interviews can be adjusted in time according to the actual situation of the interview, and there is no strict interview outline. The main purpose of the investigation is to sort out the evaluation of risk events by experts in the process of metro station construction to form expert experience samples, to analyze the relationship between risk events and identified influencing factors, and to provide case samples for safety risk prediction of metro construction. Therefore, the structured interview method of experts filling in the questionnaire is adopted in this research to carry out the investigation. In order to ensure the reliability of the collection of safety risk cases in metro construction, experts (project employers, contractors, and supervisors) who were engaged in long-term safety management works in metro construction were invited to fill out the questionnaire. All of experts had over 10 years of working experience and participated in more than five metro station construction projects. The above research provides sample data for subsequent construction safety risk-prediction research.

### 3.3. Stage 3: Construction of the PSO–SVM Prediction Model

**Figure 5:**
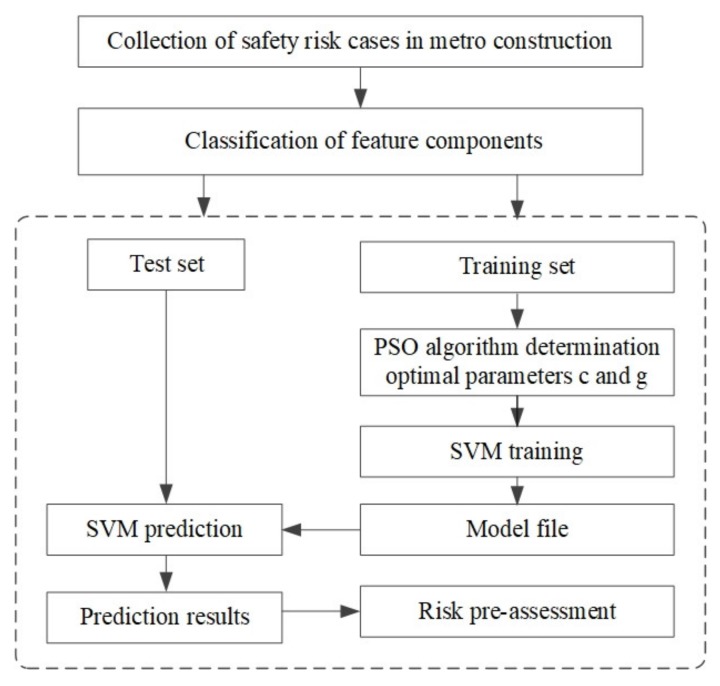
The PSO–SVM prediction model flow of safety risk for metro construction.

The establishment of the safety risk-prediction model of metro construction mainly includes training and testing. Firstly, safety risk cases are collected as sample data; then, the processed data samples are selected randomly as training sets, and the remaining samples are used as test sets. Secondly, the PSO algorithm is used to optimize the model parameters, which can be derived according to Equation (9), and the training set is used to learn the SVM model. Finally, the accuracy of the prediction ability of the model is tested by comparing the test results with the original data. The flow chart of the safety risk intelligent prediction model of metro construction is shown in Figure 5.

### 3.4. Stage 4: The Safety Risk Prediction of Metro Station Construction

If the training sample classification accuracy of the constructed PSO–SVM prediction model is relatively high, it shows that the PSO–SVM model has a relatively high accuracy for the training set and the testing set model. The PSO–SVM model can make a more scientific prediction for the safety risk of metro construction. According to the sample classification accuracy of Stage 3, it is determined whether to use the PSO–SVM prediction model constructed in this paper to predict the safety risk of the specific metro station construction project.

## 4. Case Study

### 4.1. Determination of Influencing Factors of Safety Risk in Metro Station Construction

The code of construction safety management is a summary of construction safety management after years’ experience. Therefore, code reading and review is an effective way to acquire factors (see Table 1). The main code of construction safety management issued by mainland China were mainly used in this research, and the scope was appropriately expanded; meanwhile, the relevant laws and regulations of Hong Kong, Singapore, Japan, and other regions or countries were referenced as shown in Table 1. Then, based on the in-depth analysis of the relevant literature on the influencing factors of safety risk in construction (especially metro engineering), the influencing factors that were generally considered to be more important in most studies were extracted, as shown in Table 2.

**Figure 6:**
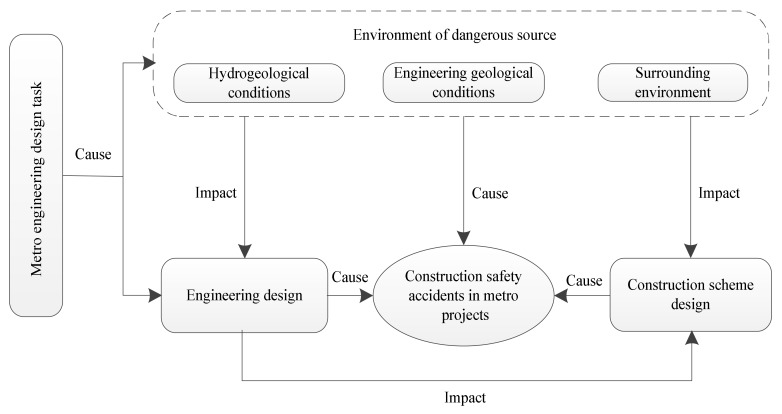
Association graph of research content of Design for Safety (DFS).

Hydrogeology, engineering geology and surrounding environment are important basic data in the process of metro construction. The uncertain factors such as complex surrounding environment, hydrogeological conditions, and engineering geological conditions constitute the dangerous source environment of the construction safety risk, which is the original factor impact of the safety risk of metro construction [76]. Because the dangerous source environment provides technical parameters for design scheme, the uncertainty of environment of dangerous sources will have an impact on the engineering design and the construction scheme design. In addition, if the dangerous source environment is handled improperly in the construction process, it will directly cause safety accidents. The main basis of the metro construction process is the design scheme. It can be said that the safety hazard is also designed [77]. Therefore, engineering design defects or errors will directly cause safety risks. According to the process of engineering design → construction scheme design → implementation scheme, engineering design is the basis of construction scheme design. Therefore, the engineering design will have an impact on construction scheme design. The relationship between the factors is shown in Figure 6. Accordingly, influencing factors were identified from the dangerous source environment, project design scheme and construction scheme design in this research. According to these three dimensions, the influencing factors of safety risk were identified. Therefore, the influencing factors of metro construction safety risks are shown in Table 2.

### 4.2. Collection of Safety Risk Cases in Metro Construction

During the interview process, in order to ensure that the experts can make more accurate judgments on the content of the interviews, the content of the options were depicted and described in the questionnaire, and an expert judgment reference was designed. For example, the respondent can make a judgment on the construction safety risk level according to the risk-level standard in the “Guidelines for Risk Management of Urban Rail Transit Underground Engineering Construction” (GB50652-2011), as shown in Table 3.

A Likert five-point system was used to measure safety risk influencing factors, and a corresponding judgment basis was designed. The corresponding judgment basis for the influencing factors that can be quantitatively measured was referenced. According to the rules on the relationship between the safety level of the foundation pit and the thickness of the soft soil layer [91,92], the measurement basis of influencing factors on the soft soil layer thickness is shown in Table 4.

For qualitative influencing factor indicators, the judgment basis can be formulated according to the meaning of the indicators. For example, if the design scheme of monitoring and measurement fully meets the monitoring layout, monitoring accuracy and design requirements, a score of 5 will be given; otherwise, a score of 1 will be given, as shown in Table 5.

Because the interviewees directly affect the reliability, comprehensiveness and effectiveness of safety risk cases obtained for the metro construction, only interviewees who have at least 10 years of safety management experience in metro construction projects were included when selecting interviewees. The interview questionnaire was divided into three parts: the first part was the basic information of the interviewees, including the work unit, present assignment, professional title, educational background, years engaged in metro construction and project location, etc.; the second part was that interviewees who had participated or interviewees who were participating made a judgment on safety risk events of the metro station construction, such as risk type and risk level; and the third part was that interviewees made a judgment on the related safety factors aimed at construction safety risk events—for example, whether the hydrogeological condition and the selection of a support scheme were reasonable.

After the structured questionnaires of 70 experts were sorted, it was determined that there were two questionnaires in which experts believed that the safety risk events had nothing to do with the influencing factors identified. Excluding the two questionnaires, a total of 68 case samples were obtained. The safety risk categories and grades in metro station construction are shown in Table 6.

### 4.3. Construction of the PSO–SVM Model

As mentioned above, a total of 68 case samples were collected in the construction of the safety risk-prediction model of the foundation pit instability damage of metro station engineering. In this case study, 57 group samples were selected randomly as training samples and 11 group samples were used as testing samples to train and test the PSO–SVM model. The sample data are shown in Table 7.

According to the sample data of the base pit instability and safety risk-prediction model in Table 8, the MATLAB (2014b) (MathWorks, Natick, USA) and LIBSVM (Version 3.22) toolbox were used to implement the SVM model according to the construction process. When the PSO–SVM algorithm program was written, the initial parameters of the model were set. The 2011 Standard PSO with 20 particles and 50 iterations was used [93]. In this paper, the feasible range of value was extended, and the value of particles was set as 20 and the maximum iteration number was $k_{\max}=100$. Because of a lack of references of optimal g and c, the value range should be enlarged [94]. So the value range of g was $g\in \left[2^{-8},2^{8}\right]$ and c was [0.1,100]. In PSO, the learning factor was a random number between 0 and 2. In this paper, the learning factors were $c_{1}=1.5$,$c_{2}=1.7$ [95]. In addition, the criterion for parameter evaluation was the minimum root mean square error (MSE). The machine environment was a i5-2430M central processing unit (CPU) 2.40 GHz, with 4.0 GHz memory and running on the Windows 7 operating system. After initialization, the calculation program was processed to read the sample data of the training set. The penalty parameter c=26.70 and kernel parameter g=0.039 of SVM were obtained. 

**Figure 7:**
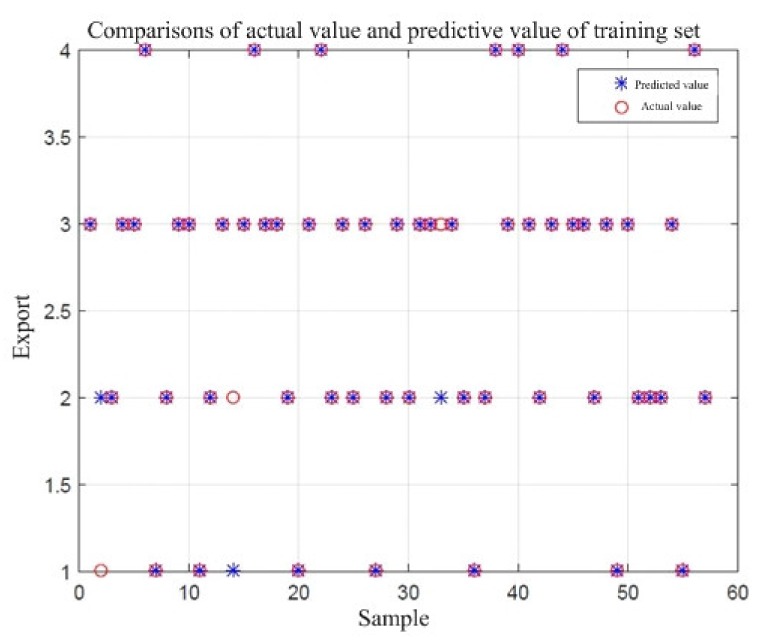
Comparisons of actual values and predicted values of the training set.

**Figure 8:**
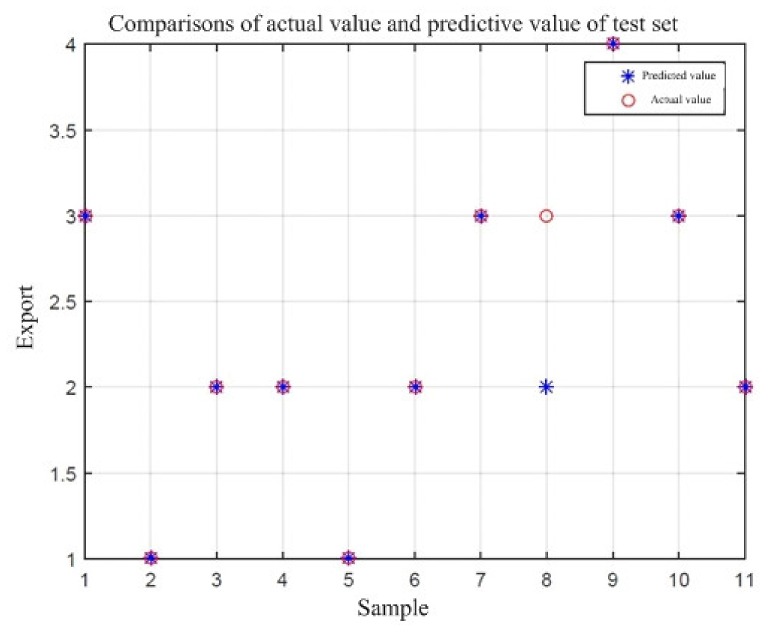
Comparisons of actual values and predicted values of the test set.

The sample data of the foundation pit instability failure category were put into the LIBSVM toolbox in the MATLAB program; then, the optimal (c,g) parameter combination was searched by the PSO, and 57 randomly selected sample data were trained to obtain a training model. The comparison between the actual and predicted values in the training set is shown in Figure 7. In Figure 7, x-axis represents the sample size of training set, and y-axis is the class value of safety risk level. Then, the regression prediction was made for the test data of 11 groups of randomly selected test samples. The comparison between the actual value and the predicted value is shown in Figure 8.

From the results of the operation in Figure 7, it can be found that, among the 57 training samples, three predicted values of samples do not overlap with the actual value. Therefore, the classification accuracy of the training samples of the SVM prediction model after parameter optimization is 94.74% (54/57). From the results of the operation in Figure 8, it can be found that in the 11 test samples, the predicted value and the actual value of sample 8 do not overlap. Therefore, the classification accuracy of the test sample is 90.91% (10/11). This shows that both the training set and the test set model have high accuracy. The PSO–SVM model can make a scientific prediction on the safety risk level of the foundation pit instability damage. On the other hand, the influencing factors in the model are all from the statistical analysis of expert knowledge, which also verifies the rationality of the integration of expert knowledge in the SVM prediction model.

### 4.4. Safety Risk Prediction of the Metro Station Construction

Station D was the transfer station of lines 1 and 2 of urban rail transit in a Chinese city. The main length of the station was 683.1 m, the width of the standard section was 41.30 m, the maximum width was 52 m, the buried depth of the structural floor was about 17.02 m, and the thickness of the roof was about 3.0 m. It was a two-story double island station on the ground floor. The total construction area of the station was 58,150 m^2^.

This study predicted the safety risk of the project in the construction preparation process of station D. Five safety managers, including the project leader and the safety supervisor of the construction unit, the chief project manager, the safety supervisor of construction enterprise and the safety director of supervision enterprise, referred to the quantitative standard of the influencing factors to quantify the two influencing factors of the metro station (the stability of the foundation pit and the safety risk of the construction). According to the comprehensive expert opinions, the quantitative results of safety risk influencing factors are shown in Table 8.

From the comparison of Figure 7 and Figure 8, it can be found that the prediction accuracy of the PSO–SVM model is high. In order to overcome the influence of SVM model random samples in this research, the original case samples were repeated under random samplings. Then different random training samples were constructed, and the SVM model was used to observe the difference of test accuracy. In this paper, 57 case samples were selected randomly as training data, the remaining 11 case samples were selected as verification data. The accuracy of test sets based on 10 random samplings are shown in Table 9. It can be found that the accuracy of the training and test sets of the PSO–SVM model has a high predictive accuracy for non-linear relationship and small samples. Meanwhile, the relevant safety risk influencing factors (Table 8) were loaded into the prediction model, and the calculation results are shown in Table 9. From Table 9, it can be found that the prediction of instability and damage of the foundation pit of station D is predicted to be a class II of safety risk, which is relatively high.

### 4.5. Results and Discussion

#### 4.5.1. Single-Factor Dynamic Adjustment Analysis

From the results of influencing factors of instability and failure safety of the engineering foundation pit, it can be found that the quantitative results of poor geological distribution (C2) and construction precipitation design (C10) are 2, and the quantitative result of the excavation depth of the foundation pit (C6) is 1, which are relatively low. Under the condition that the results of other influencing factors remain unchanged, the three factors with lower adjustment values were adjusted continuously. Then, the PSO–SVM prediction model was used to calculate the change of safety risk-prediction level. The complex coupling relationship between influencing factors and construction safety risk was uncovered and the most important influence on construction safety risk factors was identified, as shown in Table 10.

From Table 10, it can be found that under the condition that the quantitative results of other influencing factors remain unchanged, the safety risk of instability and failure of the foundation pit of the station project does not change after the quantitative results of poor geological distribution (C2) are adjusted from the original value 2 to 3. After the quantitative results are adjusted from the original value of 2 to 4, the level is reduced from level II to level III. The quantitative result of the excavation depth of the foundation pit (C6) is adjusted from the original value of 2 to 3 and 4, which does not affect the safety risk of foundation pit instability and failure. The quantitative result of the construction precipitation design (C10) is adjusted from 2 to 3, and the safety risk level of foundation pit instability and failure is reduced from II to III. 

#### 4.5.2. Multi-Factor Dynamic Adjustment Analysis

In this study, the three influence factors of poor geological distribution (C2), excavation depth of foundation pit (C6) and construction precipitation design (C10) are used to study the change of construction safety risk-prediction level under different combinations. The calculation results are shown in Table 11.

It can be seen from Table 11 that, in the C2 + C6 combination, adjusting the C2 quantification result from the original value 2 to 3 and adjusting the C6 quantification result from the original value 1 to 2, the foundation pit instability and failure safety risk of the station project do not change. If we adjust the quantitative results to 4 and 2, respectively, the safety risk level of foundation pit instability and failure is reduced from level II to level III. In the combination of C2 + C10, adjusting C2 and C10 from the original value of 2 to 3, the safety risk level of foundation pit instability and failure is reduced from the original level II to level III. In the C6 + C10 combination, adjusting the C6 quantification result from the original value 1 to 2 and adjusting the C10 quantification result from the original value 2 to 3, the safety risk level of foundation pit instability and failure is reduced from level II to level III. If we adjust the quantitative results to 3 and 4, the safety risk level of foundation pit instability and failure is reduced from the original level II to level III. Therefore, the combination of poor geological distribution (C2) and construction precipitation design (C10) is most favorable to reduce the construction safety risk level of the station, while the combination of poor geological distribution (C2) and excavation depth of foundation pit (C6) is most unfavorable to reduce the construction safety risk. The most effective way to reduce the construction safety risk is to design a reasonable construction precipitation scheme.

## 5. Conclusions

Design of Safety (DFS) is one of the most effective ways to consider safety risks in the design process, which is regarded as a risk-prevention technique for metro station construction. In order to improve the effectiveness of the application of DFS in metro station construction, it is useful to incorporate the risk-prediction procedure into the DFS. Therefore, a comprehensive framework is proposed by using the PSO–SVM model to predict the safety risk of metro station construction in this study, which provides a valuable guideline for safety risk prediction in metro station construction and provides a useful reference for engineers and managers in the design process. Firstly, 12 influencing factors related to the safety risk of metro construction are identified by using the literature review and code of construction safety management analysis. Then, the structural interview method is used to collect the safety risk cases of metro construction. Next, the PSO–SVM model is presented to predict safety risk in metro construction, in which the multi-class SVM prediction model with an improved binary tree is designed. Finally, an illustrative example is used to demonstrate the efficiency of the proposed PSO–SVM approach. 

In this study, the classification accuracy of the training samples constructed by the PSO-SVM prediction model is 94.74% (54/57), and the classification accuracy of the test samples is 90.91% (10/11), which show that the training set and the test set models all had high accuracy. In order to overcome the influence of SVM model random samples, the original case samples were repeated under random samplings. Then different random training samples were constructed, and the SVM model was used to observe the difference of test accuracy. The result of test sets based on 10 random samplings was respectively: 81.82%, 90.91%, 71.73%, 90.91%, 100.00%, 81.82%, 90.91%, 71.73%, 81.82%, 90.91%. It can be found that the accuracy of the test sets of the PSO–SVM model has a high predictive accuracy for a non-linear relationship and small samples. In addition, the relevant safety risk-influencing factors were loaded into the PSO–SVM model. The result shows that the foundation pit of station D is predicted to be a class II safety risk, which is relatively high. Meanwhile, after the computation of single and multiple factor analyses, the complex coupling relationship between influencing factors and construction safety risk was uncovered. According to the prediction results, the most important influencing factors to reduce the safety risk of metro station construction were identified, which provides a guideline for the safety risk prediction of metro construction for design process. 

Further study will be focused on the following directions: (1) the safety risks of metro construction were mainly focused on the instability of foundation pits in metro station projects in this study, which cannot cover all possible accident types in the process of metro construction. Other types of safety accidents should be investigated and analyzed in future research. (2) The intelligent safety risk prediction of the metro construction in the design process is a relatively new area of research. It is necessary to perform a more in-depth analysis of the influencing factors; for example, the technical parameters in the design process.

## Figures and Tables

**Table 1:** Codes of construction safety management in different countries or regions.

Country/Region	Code	Abbreviation
Mainland China	Standard for Construction Safety Assessment of Metro Engineering (GB50715-2011)	GB 50715
	Code for Risk Management of Underground Works in Urban Rail Transit (GB50652-2011)	GB 50652
	Code for Construction Company Safety Management Criterion (GB50656-2011)	GB 50656
	Standard for Construction Safety Inspection (JGJ59-2011) Administrative Regulations on Safety in Construction Project (Regulation No.393 of the State Council)	JGJ59 No.393
Hong Kong, China	Factories and Industrial Undertakings Ordinance (FIUO-Cap.59)	FIUO
	Occupational Safety and Health Ordinance (OSHO-Cap.509)	OSHO
Japan	Construction Occupational Health and Safety Management System (COHSMS)	COHSMS
	Guidelines and COHSMS External System Evaluation	
Singapore	The Factories (Building Operations and Work of Engineering Construction) Regulations	BOWES
	Code of Practice for Safety Management System for Construction Worksites (Singapore standard CP79:1999)	CP79

**Table 2:** Influencing factors of safety risk in metro station construction.

Dimensions	Factors	Descriptions	Sources
Dangerous source environment	C1 Distribution and water enrichment of aquifers	The uncertainty of aquifer distribution and water yield analysis brings hidden danger to the safe construction of a metro station.	GB 50652 GB 50715 [49,78,79]
	C2 Poor geological distribution	During the metro construction, special soil and poor geological conditions may be encountered, which will have a great impact on the safety of construction.	GB 50652, GB 50715 [46,79]
	C3 Soft soil thickness	The soft soil thickness is the internal cause of deep foundation pit accidents, which will cause the large deformation and displacement of the deep foundation pit.	GB 50652 GB 50656 FIUO [78,80,81,82]
Project design scheme	C4 Engineering design defects or errors	The quality of the designer determines the rationality of the design scheme and thus determines the size of the safety risk in the design results.	GB 50652 OSHO [83,84,85]
	C5 Selection of construction method	Different station types have different construction methods. Improper selection of construction methods will cause construction safety risks.	GB 50652 GB 50715 CP79 [83,86,87,88]
	C6 Excavation depth of foundation pit	With the continuous increase of the excavation depth of the foundation pit, the environment, geology and hydrological conditions will become increasingly complicated.	GB 50652 GB 50715 OSHO [83,87]
	C7 Enclosure structure design	The design of the envelope structure is the temporary or permanent structure to resist the unfavorable external environment in the process developing underground space.	GB 50652 GB 50656 [83,89]
	C8 Support system design	The support system is the temporary structure which resists the internal or external deformation of the enclosure during the excavation of the foundation pit, which is one of the main causes of safety accidents.	GB 50652 JGJ59 [86,89,90]
	C9 Safety design handover	To allow the parties to learn the engineering design for the main idea, the design basis and the construction difficulties, the designer should submit the design documents.	GB 50652 GB 50656 [76,86,90]
Construction scheme design	C10 Construction precipitation design	Groundwater is the most prominent influencing factor of engineering risk. Water-free operation of underground engineering is an important guarantee of construction safety.	JGJ59 No.393 Regulations GB50656 GB 50715 [83,86,90]
	C11 Excavation scheme design	The construction safety risks caused by different excavation methods vary greatly.	GB 50656, GB 50715 COHSMS [83,86]
	C12 Monitoring and measuring scheme	Monitoring and measurement are performed to observe and analyze the change of rock and soil characters, the deformation of the supporting structure and the surrounding environment in excavation and underground construction.	GB 50656, GB 50715 BOWES [86,89,90]

**Table 3:** Risk-level standard of metro construction project.

Probability Class		Loss Level				
		Disastrous (A)	Very Serious (B)	Serious (C)	Considerable (D)	Ignorable (E)
>0.1	Frequent	I	I	I	II	III
0.01–0.1	Possible	I	I	II	III	III
0.001–0.01	Unmeant	I	II	III	III	IV
0.0001–0.001	Infrequent	II	III	III	IV	IV
<0.0001	Impossible	III	III	IV	IV	IV

**Table 4:** The measurement criteria of soft soil layer’s thickness.

Measurement Score	1	2	3	4	5
Soft soil thickness	>5 m	4 m <h≤ 5 m	3 m <h≤ 4 m	2 m <h≤ 3 m	h≤ 2 m

**Table 5:** The measurement criteria for monitoring programs.

Measurement Score	Monitoring and Measuring Design Scheme
1	No monitoring and measurement design or serious non-compliance
2	Inconformity
3	Basically consistent
4	More consistent
5	Fully consistent

**Table 6:** Classifications and grade statistics of safety risks in metro station construction.

Risk Category	Risk Level			
	I	II	III	IV
Instability and failure of foundation pit	10	21	29	8

**Table 7:** The sample data for the instability failure of the foundation pit.

Sample	Influence Factor												Risk Level
	C1	C2	C3	C4	C5	C6	C7	C8	C9	C10	C11	C12	
1	4	3	5	3	4	4	5	3	3	4	4	4	III
2	2	1	2	2	4	1	3	3	2	3	3	3	II
3	5	4	4	4	5	4	5	5	4	5	5	5	IV
4	4	3	3	3	4	4	5	3	3	4	4	5	III
5	3	3	1	4	4	2	4	3	3	3	4	2	II
6	1	1	2	4	4	2	3	4	2	2	3	3	I
7	3	1	3	3	4	2	3	3	4	2	5	2	II
8	4	3	5	4	5	4	4	4	4	4	5	4	III
9	2	2	2	4	4	1	4	2	2	1	4	2	I
10	2	1	1	4	3	2	3	3	4	2	5	3	II
11	4	2	4	4	5	3	4	4	4	4	5	4	III
12	4	5	5	4	5	4	5	5	4	5	5	4	IV
13	4	1	1	2	3	3	3	4	2	1	3	3	II
14	4	3	4	3	4	5	4	3	4	4	5	5	III
15	3	4	4	4	5	2	3	4	3	3	5	3	III
16	3	1	1	2	3	1	2	3	3	1	3	1	I
17	2	2	1	4	4	2	3	5	3	3	4	3	II
18	2	1	2	3	4	2	3	2	3	2	3	3	II
19	1	2	1	3	3	2	3	3	1	2	3	1	I
20	2	3	2	4	3	1	4	4	3	2	3	4	III
21	1	1	2	4	3	1	3	3	2	2	3	2	I
22	3	2	3	3	3	1	4	4	3	2	3	4	II
23	5	4	4	5	5	3	5	4	5	4	5	5	IV
24	3	3	4	5	5	3	4	3	4	5	5	4	III
25	2	2	1	3	4	2	4	4	3	3	4	3	II
26	2	1	1	4	4	3	4	3	3	3	4	3	II
27	2	4	3	4	5	4	5	5	4	4	5	5	III
28	3	4	4	4	5	4	3	4	5	3	4	5	III
29	3	1	2	3	3	1	4	4	3	2	5	4	II
30	3	4	3	5	5	3	3	4	3	4	5	4	III
31	2	1	2	2	4	1	3	4	3	2	3	3	I
32	3	1	3	3	3	1	4	4	4	1	3	4	II
33	5	5	5	4	5	4	5	5	4	5	5	4	IV
34	2	3	5	5	5	2	4	3	5	5	4	4	III
35	5	3	5	5	4	2	5	4	5	4	4	5	III
36	3	3	4	5	4	2	4	4	4	4	5	4	III
37	3	3	1	4	4	2	4	3	3	3	4	2	II
38	2	3	5	4	5	3	4	4	5	5	4	4	III
39	4	4	4	4	5	4	5	5	4	5	5	5	IV
40	2	2	1	3	4	2	4	4	3	3	4	3	II
41	3	3	4	4	4	2	5	4	4	3	5	5	III
42	2	2	1	4	4	3	4	3	3	2	4	2	II
43	2	3	5	4	5	3	5	3	5	5	4	4	III
44	2	1	2	4	4	1	3	4	2	2	3	2	I
45	3	2	2	4	3	1	4	4	3	2	3	4	II
46	4	5	5	4	5	4	5	5	5	5	4	4	IV
47	2	1	2	4	4	1	3	4	2	2	4	2	I
48	4	5	5	4	5	4	5	5	4	5	5	5	IV
49	3	1	1	4	4	2	3	4	2	1	4	3	II
50	1	1	2	3	3	1	5	4	3	2	3	2	II
51	3	3	4	3	4	4	5	3	4	4	5	5	III
52	2	2	3	3	4	2	4	3	3	2	4	3	I
53	4	1	1	3	3	1	3	4	4	2	3	3	II
54	4	3	4	3	4	4	5	3	3	4	4	5	III
55	5	2	5	5	4	4	5	5	5	4	4	5	III
56	4	4	4	4	5	4	5	5	5	5	5	5	IV
57	3	3	4	5	5	3	4	3	4	5	5	4	III
58	1	2	2	4	3	1	4	4	3	2	3	4	II
59	3	3	4	4	4	2	3	4	4	3	4	4	III
60	4	3	4	3	5	3	4	3	3	5	4	4	III
61	4	4	3	3	4	3	5	3	3	5	4	5	III
62	2	1	2	3	3	1	2	4	2	3	3	3	I
63	2	3	1	4	4	2	4	3	3	3	4	2	III
64	3	3	3	5	5	2	3	5	3	4	5	4	III
65	2	2	2	3	3	1	4	4	3	2	3	3	II
66	2	3	5	5	5	3	4	3	5	5	4	4	III
67	5	4	5	4	5	4	5	5	5	5	4	4	IV
68	4	3	4	5	4	3	4	4	5	4	5	5	III

**Table 8:** The quantitative results of influencing factors on the safety risk of station D.

Factor	C1	C2	C3	C4	C5	C6	C7	C8	C9	C10	C11	C12
Quantitative results	3	2	4	4	4	1	3	4	4	2	4	3

**Table 9:** Results of accuracy and predicted risk level.

Random Number (RN)	RN 1	RN 2	RN 3	RN 4	RN 5	RN 6	RN 7	RN 8	RN 9	RN 10
Training sets	92.98%	89.47%	94.74%	85.97%	89.47%	91.22%	94.74%	91.22%	89.47%	94.74%
Test sets	81.82%	90.91%	71.73%	90.91%	100.00%	81.82%	90.91%	71.73%	81.82%	90.91%
Risk level	II	II	II	II	II	II	II	III	II	II

**Table 10:** Level changes of safety risk prediction by single factors.

Influence Factor	Quantitative Result Adjustment	The Risk Level of Foundation Pit Instability and Failure
C2	3 (+1)	II
	4 (+2)	III
C6	2 (+1)	II
	3 (+2)	II
C10	3 (+1)	III

**Table 11:** Level changes of safety risk prediction with multiple factors.

Influence Factor	Quantitative Result Adjustment	Foundation Pit Instability and Failure Risk Level
C2 + C6	C2 (+1) = 3 + C6 (+1) = 2	II
	C2 (+2) = 4 + C6 (+1) = 3	III
C2 + C10	C2 (+1) = 3 + C10 (+1) = 3	III
	C2 (+2) = 4 + C10 (+2) = 4	III
C6 + C10	C6 (+1) = 2 + C10 (+1) = 3	III
	C6 (+2) = 3 + C10 (+2) = 4	III
